# Emerging Role of Serum Glucocorticoid-Regulated Kinase 1 in Pathological Pain

**DOI:** 10.3389/fnmol.2021.683527

**Published:** 2021-05-20

**Authors:** Baowen Liu, Ningbo Li, Zhigang He, Xianwei Zhang, Guangyou Duan

**Affiliations:** ^1^Department of Anesthesiology, Tongji Hospital, Tongji Medical College, Huazhong University of Science and Technology, Wuhan, China; ^2^Department of Emergency Medicine, Tongji Hospital, Tongji Medical College, Huazhong University of Science and Technology, Wuhan, China; ^3^Department of Anesthesiology, The Second Affiliated Hospital, Chongqing Medical University, Chongqing, China

**Keywords:** serum glucocorticoid-regulated kinase 1 (SGK1), inflammatory pain, neuropathic pain, emotion-related pain, opioid tolerance

## Abstract

Currently, the management of acute and chronic pain in clinical practice remains unsatisfactory due to the existence of limited effective treatments, and novel therapeutic strategies for pathological pain are urgently needed. In the past few decades, the role of serum and glucocorticoid-inducible kinase 1 (SGK1) in the development of pain and diurnal rhythms has been implicated in numerous studies. The expression levels of *SGK1* mRNA and protein were found to be elevated in the spinal cord and brain in various pathological pain models. Blocking SGK1 significantly attenuated pain-like responses and the development of pathological pain. These studies provide strong evidence that SGK1 plays a role in the development of various types of pathological pain and that targeting SGK1 may be a novel therapeutic strategy for pain management. In this review article, we provide evidence from animal models for the potential role of SGK1 in the regulation of pathological pain caused by inflammation, nerve injury, psychiatric disorders, and chronic opioid exposure.

## Introduction

Pain is the most common reason a patient seeks medical care and is a major health concern for humans (Mäntyselkä et al., 2001; Gereau et al., 2014). Pain that lasts more than 3 months is defined as chronic pain, which is divided into chronic primary pain and chronic secondary pain in the 11th edition of* the International Classification of Diseases* (Treede et al., 2019). Secondary pathological pain can be categorized as inflammatory pain, neuropathic pain, postsurgical pain, or cancer pain. The management of chronic pain remains unsatisfactory, and the development of novel therapeutic strategies need to better understand the underlying mechanisms. In recent years, it has been suggested that serum and glucocorticoid-inducible kinase 1 (SGK1) may represent a potential target in the regulation of pathological pain (Géranton et al., 2007).

*SGK1* was first identified in 1993 as a serum and glucocorticoid-sensitive gene by Webster et al. (1993) in a rat mammary tumor cell. It belongs to the protein kinase (PK) A/PKG/PKC family (Webster et al., 1993; Lang et al., 2006; Pearce et al., 2011). Subsequently, human *SGK1* was identified by Waldegger et al. (1997) as a cell volume-sensitive gene that was upregulated by cell shrinkage. SGK2 and SGK3, two isoforms of the SGK family, were identified by Kobayashi et al. (1999). SGK1 and SGK3 are ubiquitously expressed in the central nervous system (CNS). In contrast, SGK2 is rarely detected in the brain (Kobayashi et al., 1999). It was reported that SGK2 might not be significantly involved in CNS functions since the upregulation of expression of *SGK1* and *SGK3*, but not *SGK2*, was detected following forced swim stress (Yuen et al., 2011). In addition, the potential role of SGK3 in the CNS is largely unknown because there is currently no commercial drug that antagonizes SGK3 activity. Therefore, many studies focused on SGK1.

The activation of SGK1 is involved in various signaling cascades, including 3-phosphoinositide dependent kinase 1, phosphatidylinositide-3-kinase, and mammalian target of rapamycin (Waldegger et al., 1997; Lang et al., 2018). In accordance with its name, the expression of SGK1 is sensitive to glucocorticoids and is regulated by a wide variety of stimuli, including diverse hormones, such as mineralocorticoids, gonadotropins, and various mediators such as cytokines and growth factors (Chen et al., 1999; Gonzalez-Robayna et al., 2000; Lang and Cohen, 2001). Recently, it has also been demonstrated that the expression of SGK1 is further affected by ischemia, mechanical stress, DNA damage, oxidative stress, neuronal injury, and neuronal excitation (Lang et al., 2006, 2009; Lang and Voelkl, 2013). Furthermore, SGK1 is a serine-threonine protein kinase which is involved in a broad range of physiological functions such as cellular glucose uptake and glycolysis, cell survival, cell migration, angiogenesis, and wound healing (Lang et al., 2018). SGK1 is a powerful stimulator of transcription factors such as nuclear factor kappa-B (NF-κB), cAMP responsive element-binding protein, p53 tumor suppressor protein, and activator protein-1. The most striking physiological function of SGK1 is its regulation of several channels and transporters: calcium, chlorine, potassium, and sodium channels such as epithelial sodium channel, voltage-gated potassium channel 1.3, transient receptor potential channels 5, and voltage-gated sodium channel 1.5 (Boehmer et al., 2003; Henke et al., 2004; Vallon and Lang, 2005; Marionneau and Abriel, 2015). The diversity of stimulation mechanisms of SGK1 might support the critical role of SGK1 as an intracellular regulator in cellular responses to a variety of stimulis. Recently, accumulating studies have focused on the role of SGK1 in neuronal plasticity and neuronal excitability and pain disorders. Here, we review the evidence of the involvement of SGK1 in the pathology of pain. Given that the literatures remain limited, we focused on the role of SGK1 in inflammatory pain, neuropathic pain, psychiatric disorder-related pain, and opioid tolerance.

## Functions of SGK1 in The Nervous System

**Figure 1:**
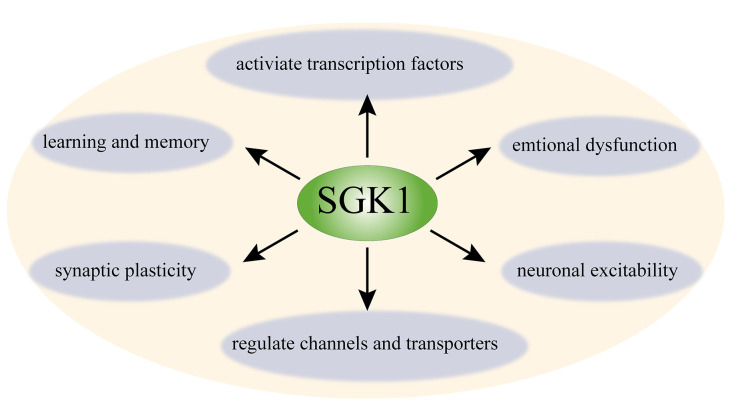
Schematic diagram of function of SGK1 in the central nerve system. SGK1 is ubiquitously expressed in the CNS. SGK1 is considered as a regulator of transcription factors (e.g., NF-κB, AP-1, and CREB) and several channels and transporters (e.g., ENaC, Kv1.3, TRPV5, and Kv7); it is also involved in neuronal excitability, synaptic plasticity, and learning, memory, and emotional dysfunction. SGK1, serum and glucocorticoid-inducible kinase 1; NF-κB, nuclear factor κ-B; CREB, cAMP-responsive element-binding protein; AP-1, p53 tumor suppressor protein and activator protein-1; ENaC, epithelial sodium channel; Kv1.3, voltage-gated potassium channel 1.3; TRPV5, transient receptor potential channels 5.

SGK1 is widely expressed in the CNS (Kobayashi et al., 1999). SGK1 is expressed in neurons and non-neuronal cells, such as astrocytes, oligodendrocytes, and a minor proportion of microglia in the CNS (Wärntges et al., 2002; Miyata et al., 2011; Peng et al., 2013; Slezak et al., 2013; Koyanagi et al., 2016). In the past decades, the function of SGK1 in the CNS has been assessed in numerous studies (Tsai et al., 2002; Nishida et al., 2004; Stichel et al., 2005; Figure 1). To date, SGK1 has been implicated in learning, memory formation, and long–term potentiation (LTP) in the hippocampus (Tsai et al., 2002; Ma et al., 2006). The expression of phosphorylated SGK1 is upregulated during LTP, and suppressing the activation of SGK1 impairs the maintenance of LTP in hippocampal neurons, suggesting that SGK1 plays a facilitating role in synaptic plasticity (Ma et al., 2006). Additionally, SGK1 has been shown to be involved in regulating spinal glutamatergic transmission (Peng et al., 2012, 2013). Furthermore, it has been shown to participate in neuronal excitability. SGK1.1, a neuronal isoform of SGK1 with higher protein stability, is highly expressed in the nervous system (Arteaga et al., 2008). Miranda et al. (2013) showed that SGK1.1 is involved in neuronal excitability as a physiological modulator of M-current, which is a voltage-dependent non-inactivating potassium conductance generated by members of the Kv7 channel family. SGK1.1 upregulated M-current in both HEK293 cells and *Xenopus* oocytes. The M-current level in neurons isolated from transgenic mice with increased SGK1.1 activity was also significantly increased (Miranda et al., 2013). Moreover, the important role of SGK1 in stress responses and emotional dysfunction has also been suggested by several studies (Yuen et al., 2011; Anacker et al., 2013). Given its function in neuronal plasticity and neuronal excitability, accumulating studies have focused on the role of SGK1 in neuronal signaling and pain disorders.

## SGK1 and Inflammatory Pain

Various studies have established the vital role of SGK1 in inflammation (Géranton et al., 2007; Kleinewietfeld et al., 2013; Binger et al., 2015; Venkatesha and Moudgil, 2019). The inflammatory signaling of cardiac, vascular, adipose, and renal tissues involves SGK1 (Borst et al., 2015; Schernthaner-Reiter et al., 2015; Gan et al., 2018; Van Beusecum et al., 2019). SGK1 responds to various inflammatory stimuli and mediates various inflammatory factors (Table 1). For example, IL-6, which is an inflammatory cytokine with wide-ranging biological effects and considered to play a vital role in the development of pathological pain (Lee et al., 2010; Guptarak et al., 2013; Baastrup et al., 2014; Fang et al., 2015), could activate SGK1 *via* p38 MAPK-dependent mechanisms (Meng et al., 2005). Additionally, SGK1 supports inflammation by activating different inflammatory-related cytokines and transcription factors. IL-17, which has potent proinflammatory properties and is predominantly produced by CD4^+^ helper *T* cells, has been demonstrated to be involved in various pain disorders by an increasing number of studies (Noma et al., 2011; Zheng et al., 2014; Isailovic et al., 2015). IL-17 knockout mice showed reduced pain behavior in inflammatory pain and peripheral nerve injury models (Cao and DeLeo, 2008; Day et al., 2014). A study by Kleinewietfeld et al. (2013) showed that SGK1 could upregulate IL-17-producing CD4^+^ helper T cells. Additionally, the expression of IL-17 was significantly decreased by silencing SGK1 using shRNA, suggesting that SGK1 plays a critical role in IL-17 related pathological processes (Kleinewietfeld et al., 2013). Moreover, it has been reported that SGK1 upregulates NF-κB, a proinflammatory transcription factor, and subsequently stimulates the expression of a wide variety of inflammatory mediators (Tai et al., 2009). However, further studies are needed to assess the direct association between these inflammatory mediators and SGK1 in pain models.

**Table 1:** Inflammatory mediators that regulate or respond to serum and glucocorticoid-inducible kinase 1 (SGK1) inflammatory pain.

Mediators		Regulate or respond to SGK1	References
Cytokines	IL-6	Regulation	Meng et al. (2005)
	IL17	Respond	Kleinewietfeld et al. (2013)
Transcription factor	NF-κB	Respond	Tai et al. (2009)
Effect protein	MeCP2	Regulation	Géranton et al. (2007)
	GRASP-1	Respond	Peng et al. (2012)
Small GTPase	Rab4	Respond	Peng et al. (2012)

**Figure 2:**
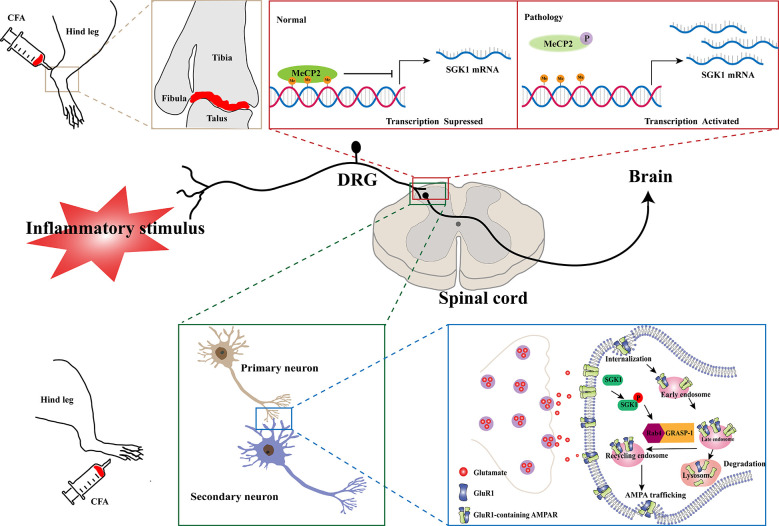
Schematic illustration of potential mechanisms of SGK1 in the development of inflammatory pain. In a normal physiological state, the expression of SGK1 is repressed by the transcriptional repressor MeCP2. In a CFA-induced joint inflammatory state, MeCP2 is found to be phosphorylated after peripheral noxious stimulation and result in activation of SGK1 gene transcription (the upper panel). The GluR1-containing AMPARs are crucially involved in processing synaptic nociceptive inputs, and the trafficking and recruitment of GluR1-containing AMPARs increase glutamatergic synaptic transmission. The spinal SGK1 phosphorylation potentiates glutamatergic synaptic transmission by promoting GRASP-1/Rab4-dependent GluR1-containing AMPARs trafficking in the CFA-induced inflammatory pain model (the lower panel). SGK1, serum- and glucocorticoid-inducible kinase 1; CFA, complete Freund’s adjuvant; MeCP2, Methyl-CpG-binding protein 2; P, phosphorylation; Me, Methyl; DRG, dorsal root ganglion; Rab4, Ras-related protein 4; GRASP-1, glutamate receptor-interacting protein associated protein-1; AMPAR, a-amino-3-hydroxy-5-methyl-4-isoxazolepropionic acid receptor.

Inflammatory pain, characterized by hyperalgesia caused by hyperexcitability of nociceptive primary afferent and dorsal horn neurons, is a common clinical symptom of inflammatory diseases. The involvement of SGK1 in inflammatory pain was first reported by Géranton et al. (2007) using a rat model of complete Freund’s adjuvant (CFA)-induced joint inflammation. In this study, using a genome-wide microarray, they found that the expression of SGK1 in the superficial dorsal horn was upregulated after inflammation was induced. Furthermore, the antagonism of spinal SGK1 by intrathecal antisense RNA perfusion significantly ameliorated inflammation-related hyperalgesia (Géranton et al., 2007). They also found that the upregulation of SGK1 might be attributed to a decrease in gene repression by the phosphorylation of methyl-CpG-binding protein 2 (MeCP2), which was increased in the dorsal horn after noxious stimulation. This evidence supports the role of SGK1 in the induction and maintenance of an inflammatory pain state (Figure 2, upper panel). Another study, revealed that spinal phosphorylated SGK1 levels were significantly increased after CFA-provoked behavioral hyperalgesia (Peng et al., 2012). Additionally, intrathecal application of GSK-650394, an SGK1 activation antagonist, prevented CFA-induced hyperalgesia and increased SGK1 phosphorylation. Furthermore, Géranton et al. (2007) demonstrated that spinal SGK1 phosphorylation potentiated glutamatergic synaptic transmission by regulating GRASP-1/Rab4-dependent GluR1-containing AMPAR trafficking in a CFA-induced inflammatory pain model (Peng et al., 2012; Figure 2, lower panel). In a subsequent study, Olango et al. found that the expression level of SGK1 mRNA was increased in the dorsal horn of the spinal cord after intraplantar formalin injection, suggesting that formalin-induced nociceptive behavior is related to the increased SGK1 mRNA expression (Olango et al., 2014). These studies suggest that the activation of SGK1 is involved in the development and maintenance of inflammatory pain and may be a novel therapeutic strategy for the treatment of inflammatory pain.

## SGK1 and Neuropathic Pain

**Figure 3:**
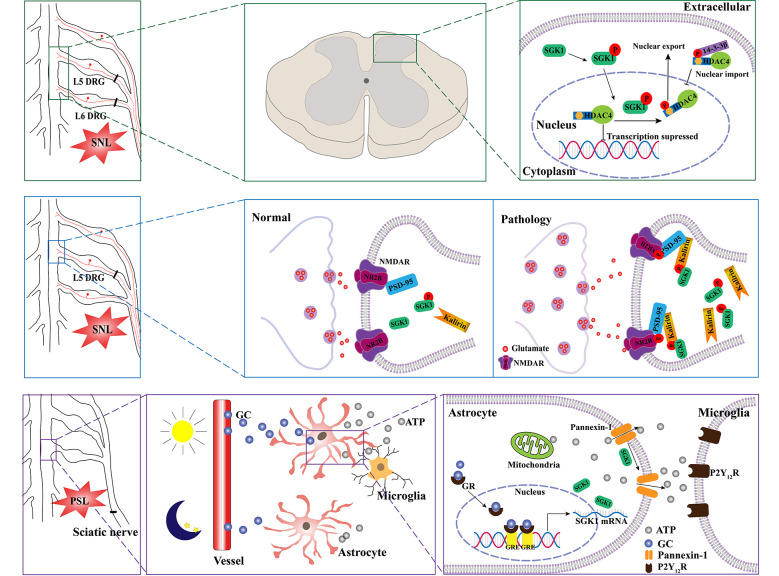
Schematic illustration of potential mechanisms of SGK1 underlying the development of neuropathic pain. SNL induces the phosphorylation of SGK1. The activation of SGK1 promotes HDAC4 phosphorylation and export from nucleus to cytoplasm by 14-3-3, which restricts HDAC4 to the cytoplasm. Therefore, the decreased inhibition of nociception-associated gene expression by HDAC4 might contribute to SNL-induced allodynia (the upper panel). The upregulation of phosphorylated SGK1 in the SNL model enhances the expression of kalirin, which provokes the phosphorylation of PSD-95 coupling NR2B subunit, resulting in the increase of glutamatergic neurotransmission (the middle panel). Diurnal alterations in pain hypersensitivity result from diurnal variations in glucocorticoid levels. The increase in glucocorticoid levels induces an upregulation of SGK1 expression in spinal astrocytes and enhances extracellular ATP release by opening pannexin-1 hemichannels. Thereafter, the released ATP binds to P2Y12 receptors and induces the activation of microglia (lower panel). SNL, spinal nerve ligation; SGK1, serum- and glucocorticoid-inducible kinase 1; HDAC4, histone deacetylase 4; P, phosphorylated; NMDAR, N-methyl-D-aspartate receptor; NR2B, N-methyl-D-aspartate receptor 2 B; PSD-95, postsynaptic density-95; PSL, partial sciatic nerve ligation; GC, glucocorticoid; GR, glucocorticoid receptor; GRE, glucocorticoid receptor responsive elements; P2Y_12_R, purinergic receptor P2Y12.

A growing number of studies have indicated that SGK1 plays a role in the pathogenesis of neuropathic pain. A previous study by Peng et al. (2013) assessed the relationship between SGK1 and neuropathic pain using a spinal nerve ligation (SNL) rat model. They found that the expression of phosphorylated SGK1 was upregulated in the dorsal horn of SNL rats. They found that blocking the activation of SGK1 pharmacologically using GSK-650394 could ameliorate SNL-induced allodynia. The kalirin/PSD-95/pNR2B cascade may be the downstream mechanism regulated by SGK1 in the development of SNL-induced neuropathic pain. Collectively, these results provide evidence that spinal SGK1 is involved in the development of neuropathic pain caused by spinal ligation (Peng et al., 2013; Figure 3, middle panel). Similar results were reported in a subsequent study by Lin et al. (2015). They showed that SNL-associated allodynia was accompanied by the activation of spinal SGK1, providing further evidence for the role of spinal SGK1 in the etiology of allodynia following nerve injury. Additionally, their results demonstrated that spinal SGK1-dependent HDAC4 phosphorylation and cytoplasmic retention, which may result in decreased nucleus-bound HDAC4 and altered nociception-associated gene expression, probably contribute to SNL-induced allodynia (Lin et al., 2015; Figure 3, upper panel). Nevertheless, the precise underlying molecular and cellular mechanisms remain unknown; these may be complicated and require further investigation.

Recently, an interesting study linked SGK1 to diurnal variations in pain hypersensitivity, which has been confirmed in patients with diabetic neuropathy, fibromyalgia, multiple sclerosis, and cancer (Solaro et al., 2000; Bellamy et al., 2004; Odrcich et al., 2006; Saini et al., 2013). Koyanagi et al. (2016) used a partial sciatic nerve ligation (PSL) mouse model, which has been shown to undergo diurnal oscillations in the threshold of mechanical allodynia. They found that diurnal alterations may be related to glucocorticoid levels (Kusunose et al., 2010). Using oligonucleotide microarray analyses, they identified the glucocorticoid-regulated gene *SGK1* as a diurnal time-dependent spinal gene. They then validated that the mRNA and protein levels of SGK1 had diurnal oscillations in the spinal cord of PSL mice, accompanied by the oscillation of glucocorticoids. Moreover, their results demonstrated that SGK1 enhanced glucocorticoid-induced ATP release from spinal astrocytes by opening pannexin-1 hemichannels, contributing to glucocorticoid-induced exacerbation of mechanical allodynia in PSL mice (Koyanagi et al., 2016; Figure 3, lower panel). Most recently, Koyrangi et al. found that sulfasalazine has inhibitory effects on SGK1, and an intrathecal injection of sulfasalazine significantly alleviated PSL-induced hypersensitivity, while an oral administration of this drug had no significant analgesic effects due to its low bioavailability and limited spinal distribution (Yasukochi et al., 2021). However, a concomitant oral administration of sulfasalazine with febuxostat, an approved drug that inhibits the xenobiotic transporter breast cancer resistance protein, improved the analgesic effects by facilitating the distribution of sulfasalazine to the spinal cord (Yasukochi et al., 2021). Given that there are no available drugs to inhibit SGK1 in the spinal cord, this study provides a strategy for the treatment of the diurnal exacerbation of neuropathic pain.

Neural precursor cell-expressed developmentally downregulated protein 4–2 (Nedd4–2), a member of the E3 ubiquitin ligase, negatively regulates many membrane proteins containing a conserved “PY motif” by increasing their rate of internalization and degradation (Abriel et al., 2000). Previous evidence has pointed to a downregulation of Nedd4–2 after a spared nerve injury (SNI), resulting in the dysregulation of Navs, which may lead to hypersensitivity associated with nerve injuries and contribute to the genesis of neuropathic pain (Cachemaille et al., 2012; Laedermann et al., 2013). It is well known that activated SGK1 is able to phosphorylate Nedd4–2 and decrease the ubiquitylating effect of Nedd4–2, suggesting that SGK1 may be implicated in the development of neuropathic pain by mediating Nedd4–2 (Boehmer et al., 2003; Snyder et al., 2004). Recently, our results showed that sustained NGF in the dorsal root ganglion upregulated the expression of Nav1.7 by activating SCK1 to phosphorylate Nedd4–2 in a chronic post-surgical pain rodent model. Moreover, the intrathecal administration of an SGK1 inhibitor could decrease the expression of Nav1.7 and alleviate post-surgical pain hypersensitivity in rats (Liu et al., 2021). Post-surgical pain is characterized by neuropathic and inflammatory characteristics (Chapman and Vierck, 2017). Taken together, these studies imply that SGK1 is involved in the pathogenesis of neuropathic pain induced by nerve injury.

## SGK1 and Emotion-Related Pain

Recently, many studies have focused on the novel role of SGK1 in stress, depression, fear episodes, and anxiety (Luca et al., 2009; Anacker et al., 2013; Li et al., 2015; Wei et al., 2016; Zhang et al., 2019). In the context of these psychiatric disorders, the expression of SGK1 has been demonstrated to be increased in animals and humans (Anacker et al., 2013; Yuan et al., 2016). There is compelling evidence that pain and negative emotional disorders are mutually influential (de Heer et al., 2014). The association between anxiety and pain is most likely bidirectional. Anxiety is a predictor of pain, whereas chronic pain is a predictor of anxiety. A possible explanation is that anxiety and pain share potential cognitive and behavioral processes (Asmundson and Katz, 2009).

Presurgical anxiety is a common problem in surgical patients. A large body of evidence implies that presurgical psychological vulnerability, such as anxiety, is a powerful risk factor for the development of postsurgical hyperalgesia (Kehlet et al., 2006; Hinrichs-Rocker et al., 2009; Scott et al., 2016). It has been reported that presurgical anxiety is related to postsurgical pain intensity, which may aggravate and prolong postsurgical hyperalgesia (Liu et al., 2015; Raichle et al., 2015). However, the underlying mechanisms remain unclear. An animal model was established to assess the mechanisms of presurgical anxiety-induced postsurgical hyperalgesia. The animal was exposed to a single-prolonged stress (SPS) procedure prior to a planter incisional surgery to induce presurgical anxiety-like behaviors (Liu et al., 2015; Sun et al., 2017; Zhang et al., 2019). It was found that glucocorticoids were involved in the development of presurgical anxiety-induced postsurgical pain (Sun et al., 2017). More recently, another study exploring the possible underlying mechanisms of glucocorticoids in presurgical anxiety-induced postsurgical pain found that SPS induced the upregulation of plasma corticosterone in rats and that the intraperitoneal application of corticosterone mimicked SPS-induced postsurgical hyperalgesia. Furthermore, they found that the expression of SGK1 and the concentration of ATP in the spinal cord were upregulated and that the inhibitor of SGK1 markedly decreased ATP release and attenuated pain-related behaviors (Zhang et al., 2019). Similarly, another study showed that chronic corticosterone exposure increased the level of phosphorylated SGK1 in a chronic corticosterone-induced mouse model of anxiety/depression (Zhang et al., 2016). These studies suggest that SGK1 may be a downstream effector of glucocorticoids in anxiety-related disorders. Moreover, glucocorticoids have been reported to be consistently increased in humans or animal models suffering from depression or stress (Anacker et al., 2011).

As mentioned above, chronic pain is also a strong risk factor for the development of negative emotions such as anxiety, depression, and stress (de Heer et al., 2014). A recent study reported that acid-sensing ion channel 1a (ASIC1a) in the basolateral amygdala participated in the development of pain and associated anxiety in a rat CFA-induced arthritis model. Using TaqMan RT-qPCR, they found that the mRNA levels of several kinases, including SGK1, were significantly altered (Aissouni et al., 2017). This study suggests that brain SGK1 is potentially involved in pain and anxiety behaviors associated with arthritis by modulating ASIC1a (Aissouni et al., 2017). Additionally, SGK1 has been reported as a potential modulator of ASIC1, and brain-specific SGK1 has been shown to mediate the expression and function of ASIC1 (Arteaga et al., 2008). Together, these studies reinforce the idea that SGK1 plays a critical role in the development of anxiety-associated disorders and anxiety-induced hyperalgesia.

**Figure 4:**
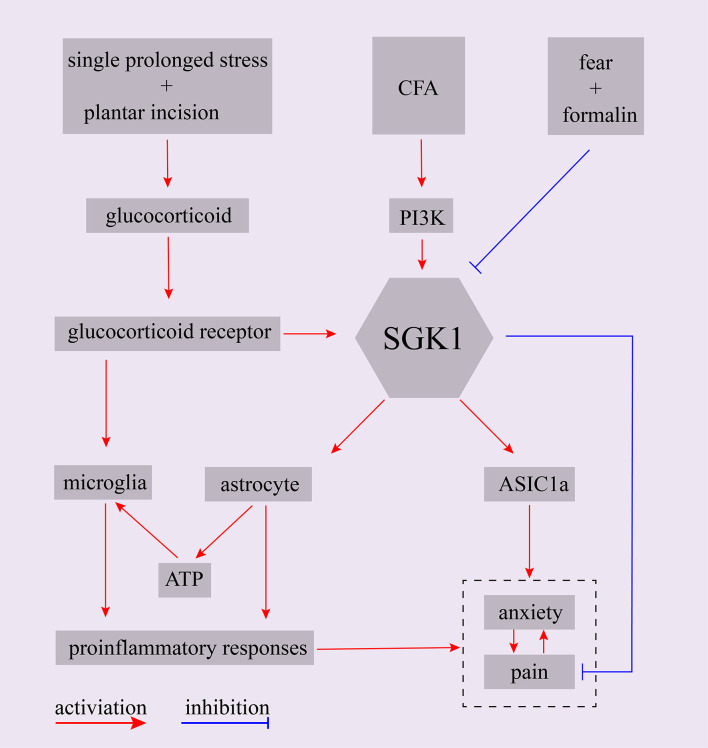
Schematic representation of potential role of SGK1 in the interaction between emotion and pain. CFA, complete Freund’s adjuvant; PI3K, phosphoinositide 3-kinase; SGK1, serum- and glucocorticoid-inducible kinase 1; ASIC1a, acid-sensing ion channel 1a.

Fear-conditioned analgesia is a phenomenon in which an animal is exposed to conditioned aversive stimuli, resulting in the profound suppression of pain responses. Olango et al. found that the expression level of *SGK1* mRNA was increased in the spinal cord of non-fear-conditioned rats following an intraplantar formalin injection. In contrast, the formalin-induced increase in *SGK1* mRNA expression, which tended to strongly decrease, was not observed in fear-conditioned rats. Although it failed to reach statistical significance, the suppression of pain-like response might be associated with the failure of formalin-induced upregulation of *SGK1* mRNA expression in fear conditions (Olango et al., 2014). A summary of the main findings of SGK1 in the interaction of emotion and pain were shown in Figure 4. These findings indicate that SGK1 should be studied when investigating the mechanisms of emotion-pain interactions, and it could be a new therapeutic target for treating emotional dysfunction in pain states or chronic pain in emotional disorders.

## SGK1 and Opioid Tolerance

Opioids have powerful analgesic effects and are widely used in clinics for the treatment of acute and cancer pain. However, chronic drug exposure unavoidably leads to many side effects, such as addiction and opioid tolerance, thereby limiting opioids’ application in chronic pain management. Chronic opioid-induced cellular alterations remain unclear. Alterations of SGK1 levels in the spinal cord and brain have been implicated after opioid administration. For example, an increase in *SGK1* mRNA was observed in the striatum of mice after acute morphine stimulation, and a selective glucocorticoid receptor antagonist attenuated the morphine-evoked upregulation of SGK1, suggesting that the alteration of SGK1 induced by morphine depends on glucocorticoid receptor signaling (Slezak et al., 2013). Furthermore, it has been reported that *SGK1* mRNA is upregulated in whole brain lysates following chronic oxycodone administration, a mu opioid receptor agonist (Hassan et al., 2010). A study by Befort et al. (2008) used a genome-wide microarray approach to analyze the transcriptional profiles of the central extended amygdala of wild-type and mu-opioid receptor knockout mice after chronic morphine. They found that chronic escalating morphine administration resulted in a robust increase in* SGK1* mRNA in wild-type mice but not in mu-opioid receptor knockout mice. However, a single injection of morphine did not alter the expression of *SGK1* mRNA. These findings indicated that SGK1 was upregulated after chronic treatment with morphine and subsequent mu-opioid receptor activation (Befort et al., 2008).

**Figure 5:**
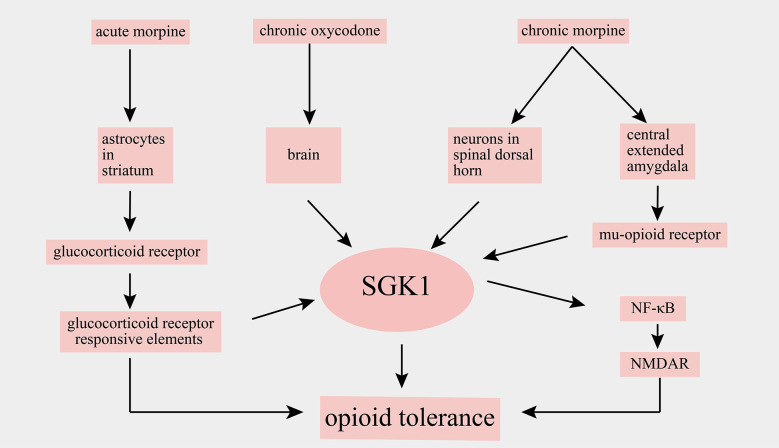
Schematic illustration of involvement of SGK1 in opioid tolerance. SGK1, serum-and glucocorticoid-inducible kinase 1; NF-κB, nuclear factor kappa B; NMDAR, N-methyl-D-aspartate receptor.

The role of SGK1 in opioid tolerance has been further defined in a recent study. In this study, Li et al. (2015) showed that the phosphorylation of SGK1 in the spinal dorsal horn was markedly increased following chronic morphine treatment and that the spinal administration of SGK1 inhibitor GSK-650394 and SGK1 small interfering RNA reduced the development of morphine tolerance by suppressing the activation of NF-κB and NMDAR (Xiao et al., 2019). A summary of the main findings of SGK1 in opioid tolerance are shown in Figure 5. In summary, these studies revealed that SGK1 may represent a potential therapeutic target for reducing opioid tolerance in the context of pain management.

## Conclusions

In this review article, we summarize the role of SGK1 in pathological pain. These studies have provided evidence that SGK1 plays a critical role in the development of inflammatory pain, neuropathic pain, emotional dysfunctions related to pain, and opioid tolerance. Treatment with the SGK1 inhibitor GSK-650394 or small interfering RNA could relieve nociceptive hypersensitivity in various pathological pain models, suggesting that SGK1 may represent a potential therapeutic target for pain management. However, there are currently no clinically approved drugs known to inhibit the activity of SGK1, and few clinical trials have studied SGK1 inhibitors in pain therapies. Although the clinical value of analgesia therapy targeting SGK1 has not been clearly confirmed, preclinical studies of SGK1will provide a basis for clinical trials and drug development. Therefore, more selective and clinically relevant drugs targeting SGK1 require further exploration in future studies and clinical trials.

## Author Contributions

BL and NL wrote and edited this review. ZH contributed in production of figures. XZ and GD drafted the manuscript. All authors contributed to the article and approved the submitted version.

## Conflict of Interest

The authors declare that the research was conducted in the absence of any commercial or financial relationships that could be construed as a potential conflict of interest.
